# Occurrence of *myo*-inositol and alkyl-substituted polysaccharide in the prey-trapping mucilage of *Drosera capensis*

**DOI:** 10.1007/s00114-017-1502-4

**Published:** 2017-09-22

**Authors:** Tetsuo Kokubun

**Affiliations:** Royal Botanic Gardens, Kew, Richmond, Surrey, TW9 3AB UK

**Keywords:** *Drosera capensis*, Carnivorous plants, Mucilage, *myo*-Inositol, Nuclear magnetic resonance spectroscopy, Bioadhesive

## Abstract

The chemical composition of the exudate mucilage droplets of the carnivorous plant *Drosera capensis* was investigated using nuclear magnetic resonance spectroscopy. The mucilage was found to contain beside a very large molecular weight polysaccharide a significant amount of *myo*-inositol. It appears that *myo*-inositol escaped detection due to the commonly applied methodology on the chemical analysis of plant mucilage, such as dialysis, precipitation of polysaccharide component with alcohol, acid hydrolysis and detection of the resultant monosaccharide (aldose) units. The possible functions of *myo*-inositol in the mucilage droplets and the fate after being washed off from the leaf tentacles are proposed. On the polysaccharide component, the presence of methyl ester and alkyl chain-like moieties could be confirmed. These lipophilic moieties may provide the prey-trapping mucilage with the unique adhesive property onto the hydrophobic insect body parts, as well as onto the nature’s well-known superhydrophobic surfaces such as the leaves of the sacred lotus plants. A re-evaluation of the mineral components of the mucilage, reported 40 years ago, is presented from the viewpoints of the current result and plants’ natural habitat. A case for re-examination of the well-studied plant mucilaginous materials is made in light of the new findings.

**Electronic supplementary material:**

The online version of this article (10.1007/s00114-017-1502-4) contains supplementary material, which is available to authorized users.

## Introduction

Ever since Darwin’s time, carnivorous plants have continued to draw interests of scholars and the public alike, and elicited imaginations once their methods of nutrient acquisitions were recognised (Chase et al. 2009; Król et al. 2012). One of the major strategies employed for capture and retention of the prey is the use of sticky ‘glues’, usually at the sites of digestion and nutrient absorption (Chase et al. 2009; Adlassnig et al. 2010; Król et al. 2012). The species of *Drosera* (Droseraceae), sundew plants, are perhaps the most widely known, featuring the flytrap leaves that bear the sticky mucilage secreted at the tips of glandular hairs (‘tentacles’). Once a prey animal lands on the leaves, the tentacles and the leaves themselves would slowly curl up to grip the prey and increase the contact area for efficient nutrient absorption (Król et al. 2012); however, the highly viscous and elastic mucilage is the primary means of prey capture and retention.

Cape sundew, *Drosera capensis* L., is endemic in the wild to the Cape Flora Region of South Africa, but is widely available for horticultural curiosities worldwide. It is a surprisingly hardy perennial plant, overwintering as dormant hibernaculum after the leaves have died back in frost. It grows, in common with a number of other carnivorous plants, well-lit, highly moist but nutrient-poor soils such as bogs. The chemical composition of its mucilage was studied and reported in a total of four articles between 1977 and 1984, together with that of *D*. *binata*, as a pure solution of high molecular weight polysaccharide (Rost and Schauer 1977; Gowda et al. 1982, 1983; Aspinall et al. 1984). Apart from the preceding cytochemical studies, these are the only studies on the chemical structures of the mucilage of *Drosera* and indeed of the family Droseraceae; otherwise, studies on the chemistry of the family are relatively limited (Schlauer et al. 2005; Egan and van der Kooy 2013). The mucilage has been explored recently as a biomimetic model of functional hydrogels that may find applications in various technological fields, including tissue engineering (Zhang et al. 2010; Lenaghan et al. 2011; Huang et al. 2015). However, a scrutiny of the original reports points to certain inconsistencies. For example, whilst a near-identical chemical structure have been proposed for the mucilage polysaccharides of *Drosera* spp. and of the stem of *Actinidia chinensis* cv. ‘Hayward’ (Actinidiaceae), the one from the former was ‘only very slowly dissolved in water’ and elastic (Rost and Schauer 1977), and that from the latter was readily soluble in water when in crude (salt) form (Redgwell 1983). Also, a ‘pure solution of polysaccharide in water’ (Rost and Schauer 1977) would not exert a sufficient binding strength for retaining a prey that attempts to free itself. An orthogonal re-examination on the composition and structure was therefore warranted, using alternative methodologies to the previous findings.

Nuclear magnetic resonance (NMR) spectroscopy is a technique to observe the chemical environment of atomic nuclei in a compound. Whilst this technique is inherently among the least sensitive spectroscopic methods, it accepts a wide range of samples as long as, generally, the sample is a clear solution and that the solution is free from a paramagnetic element or ion. Because of the non-destructive nature, richness of information and the capability to provide quantitative information when appropriate care is taken, NMR spectroscopy is being applied to a wide range of biological materials including exudates (e.g. nectar), biological fluids (e.g. urine, serum) and commercial products for detection of adulteration, ranging from food and beverages to biofuel. The mucilage of sundew is therefore amenable to direct NMR spectroscopic analysis with very minimal treatment, which is merely diluting with deuterium oxide (D_2_O). As such, the relative concentration of analytes is preserved and accurately represented by the integration of the signals (area-under-curves).

The account of this exploratory analysis is presented below. It appears that the past results were not incorrect for the employed techniques but represent only a partial composition of the whole of the mucilage; significant amounts of non-polysaccharide and potentially critical functional components were found escaped detection. Prompted by this finding, the mucilage of *Actinidia* stem was examined in the same NMR method. Further, mucilages from the root tips of *Lupinus polyphyllus* Lindl. and *Zea mays* L., the fruits of *Abelmoschus esculentus* (L.) Moench. (okra) and the leaf mesophyll of *Aloe vera* (L.) Burm.f., all of which have long history of chemical and biological studies, were also examined for comparison and for detecting the presence of previously unrecognised components.

## Material and methods

### General and reagent sources

The nuclear magnetic resonance spectra were obtained with a Bruker Avance III spectrometer at 400/100 MHz resonance frequencies for proton and carbon-13 nuclei, respectively, on a room temperature BBO probe at the sample temperature of 30 °C. Samples were dissolved or diluted in deuterium oxide (D_2_O, Aldrich, 99.9% atom D) containing 0.01% (*v*/*v*) acetonitrile (**CH**_**3**_CN) as the internal chemical shift reference at *δ*_H_ 2.06 and *δ*_C_ 1.47 ppm (Gottlieb et al. 1997). Standard Bruker microprograms were used for acquisition of 1D (^1^H, ^13^C) and 2D (COSY, HSQC, HMBC) spectra.

Laboratory reagents were obtained from commercial sources Fisher Scientific (Leicester, UK: acetonitrile, diethyl ether, isopropanol and trifluoroacetic acid) and BDH (Poole, UK: *myo*-inositol). In house-purified water with a Milli-Q Integral system (Merck Millipore, MA, US) was used throughout the laboratory experiments. Dialysis membrane tubing (cut-off MW 6000–8000) was purchased from Fisher Scientific.

### Plant material

The seeds of *Drosera capensis* L. were obtained from the retail section of the RBG, Kew, UK, and grown on a mixture of lime-free horticultural grade sand and Irish peat moss (1:1 by volume) in square plastic pots measuring 10 × 10 cm by 12 cm height. These pots were arranged three rows by six columns in deep trays (12 cm depth). All the plants used in the current study were grown outdoor for 5 to 9 years under full sunlight, and only rainwater was used for irrigation for the whole of the cultivation period. During these years, the set seeds were naturally deposited onto the growth medium and the new plants grew alongside their parents, forming a dense canopy of insect-trapping leaves. A small number of plants with white-form of the tentacles grew among the vast majorities of red forms. In winter(s) and/or periods of heavy rain, the whole plants were often immersed under water for extended time, in occasions more than 1 month continually, but were not intervened.

During the mucilage collection period (detailed below), the plants were maintained either in a well-ventilated glasshouse or an equivalent indoor environment, while ensuring maximum exposure to sunlight. The plants were provided with rainwater only at the depth of 4 to 6 cm in trays (18 pots per tray, as above). Otherwise, no artificial control was applied on the lighting or temperature throughout. The environments were practically free from insect prey. When insects were observed to have been captured in rare instances, they were removed immediately. The exudate mucilage were collected from plants in a total of 23 pots.

### Mucilage collection for NMR analysis

For initial qualitative analyses, the mucilage of *D*. *capensis* was collected by centrifugation. Leaves were excised at the base of the petiole, and the tentacle-bearing parts (‘blades’) were placed inside a 15-mL Corning tube close to the top, while the petioles were bent over and affixed to the outside of the tube with a piece of cellophane tape. The tube was centrifuged at 2050×*g* for 4 min to dislodge the mucilage. The exhausted leaves were discarded, and fresh leaves were likewise treated on the same centrifuge tube. Approximately 200 μL mucilage could be obtained from a total of 30–35 leaves. For comparison of the components, mucilages were collected from red and white forms of the leaves separately.

The collected mucilage samples were thoroughly mixed with D_2_O immediately to the final volume of 600 μL which is the required minimum volume for NMR analysis, and further centrifuged at 12,000×*g*; the viscous supernatant was carefully transferred to a 5-mm tube (Wilmad 507-PP).

### Collection of mucilage from detached leaves

Centrifugation of the leaves at a force sufficient for dislodging of the mucilages incurred a significant mechanical damage to the petioles. Therefore, 20 mature leaves, bearing visually clean mucilages without trapped prey, were excised at the base of the petiole and immersed in 40 mL water for 18 h at room temperature. The leaves were recovered from the then viscous fluid, blotted gently on tissue paper, stood individually in 15-mL vials containing approx. 10 mL water, and left under full daylight in an insect-free environment. The mucilage solution was in a meantime freeze-dried. The regeneration of the mucilages at the tips of the tentacles could be observed approximately 48 h later. The regenerated mucilages were likewise washed into water. This was repeated once more (after 72-h regeneration time), obtaining a total of three mucilage samples, which were one (A) from attached leaves and two (D1, D2) generated from the detached leaves. Thereafter, the leaves showed signs of chlorosis and/or senescence, and very little mucilage could be recovered. The three mucilage solutions A, D1 and D2 gave 14.7, 3.9 and 4.6 mg (dry wt) respectively of fibrous material after freeze-dying. For NMR analyses, 800 μL D_2_O (containing 0.01% *v*/*v* acetonitrile) was added to each of the dried mucilage and left overnight at room temperature. The whole samples were subjected to 1D proton NMR analysis without further treatment despite some turbidity.

### Estimation of the mucilage solute mass in relation to the leaf biomass

Twenty leaves with abundant mucilage on the tentacles were carefully excised at the base of petioles, lengths measured and immersed into 40 mL water in a 50-mL Corning tube. The lengths of the excised leaves were on average 6.8 ± 0.78 cm SD including the petiole, and 3.4 ± 0.30 cm SD for the leaf blade. The tube was shaken horizontally for 30 min at 120 rpm. The viscous water was decanted to a separate container and the leaves were further washed in the same manner. After four washes, the water was no longer viscous. All the washes were combined (160 mL) and freeze-dried to yield 5.9 mg of solid. The leaves were dried at 60 °C for 18 h, to a constant total weight of 132.5 mg.

### Fractionation of mucilage components

**Fig. 1:**
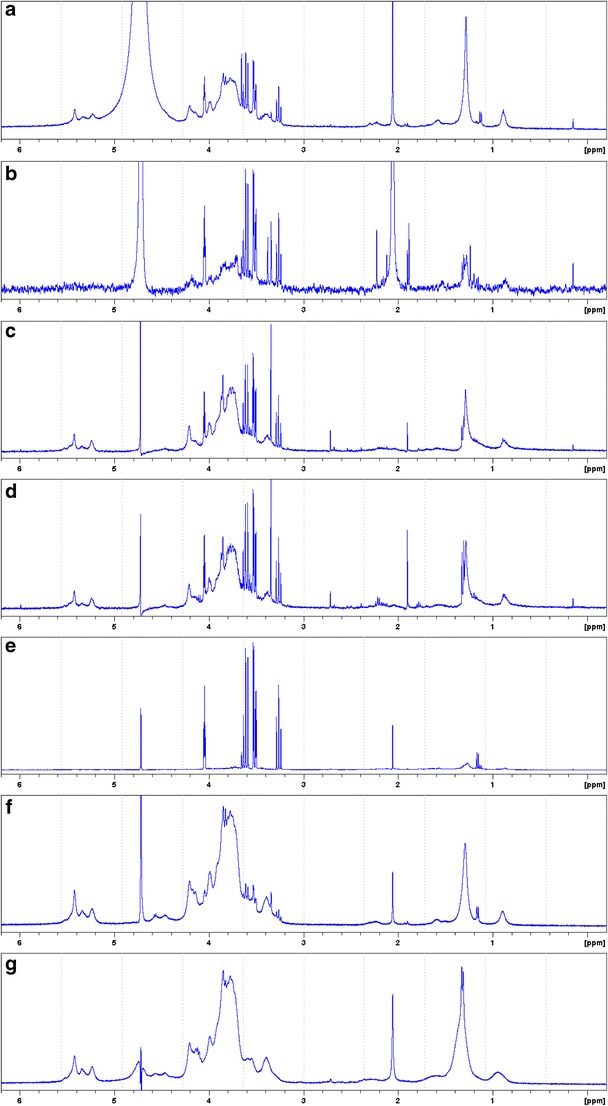
Proton NMR spectra (400 MHz, D_2_O, 30 °C) of crude mucilage (diluted, **a**–**d**) and separated mucilage components (**e**–**g**). **a** Mucilage collected by centrifugation from red tentacles. **b** Mucilage from white tentacles. **c** Regenerated mucilage from detached leaves with red tentacles (D1). **d** Regenerated mucilage from detached leaves for the second time (D2). **e** Isopropanol-water (8:2, *v*/*v*)-soluble fraction. **f** Isopropanol-water (8:2, *v*/*v*)-insoluble fraction, redissolved in D_2_O at 80 °C. **g** Retentate of dialysis, redissolved at 80 °C. Vertical axis not to scale. Chemical shifts were calibrated against the internal reference CH_3_CN at 2.06 ppm (**a**–**b**, **e**–**g**) or HDO at 4.725 ppm (**c**, **d**)

Freshly collected mucilage by centrifugation as described above, approximately 500 μL by the graduation on the 15-mL Corning tube (482.7 mg wet wt), was mixed with 1.5 mL of water with vigorous shaking and vortexing for 2 min. Eight millilitres of isopropanol was added slowly while vortexing during the whole addition period. The mixture was further vortexed for 2 min and centrifuged at 2050×*g* for 2 min. The supernatant was transferred to a 25-mL glass vial by pipetting, and the precipitate was rinsed with 2 mL diethyl ether; the diethyl ether wash was added to the supernatant fraction. Both the precipitate and supernatant were air-dried at 37 °C first, then at high vacuum (approx. 0.1 mBar) overnight, yielding 4.0 mg and 6.7 mg iPrOH-soluble and insoluble fractions, respectively. These fractions were separately dissolved in 600 μL D_2_O (with 0.01% *v*/*v* acetonitrile); heat (approx. 80 °C) was applied to the iPrOH-insoluble fraction in order to dissolve it completely (Fig. 1e, f).

The above protocol was repeated for the purpose of experiments for (1) re-mixing of the components, and (2) mixing of the isopropanol-insoluble fraction and authentic *myo*-inositol; the volumes and yields of fractions were consistent with the typical values as quoted above with respect to the amount of leaf materials used.

The removal of low-molecular weight components were also accomplished with dialysis membrane. Mucilage was collected by gently swirling 50 detached leaves in 50 mL water. The viscous solution was filtered through a 20-μm porosity polythene frit, placed in an empty solid-phase extraction cartridge (15 mL capacity; Biotage), which was in turn placed in a 50-mL Corning tube and centrifuged at 600×*g* for 1 min. The mucilage solution was then dialysed against water (4 L) at 4 °C, stirring, for a total of 46 h with six changes of water. The retentate was freeze-dried, yielding 7.6 mg of white fibrous material, which was dissolved in 800 μL D_2_O at 80 °C for NMR analysis.

### Mass collection of mucilage, fractionation and acid hydrolysis

The exudate mucilage was collected during the months of July and August 2016, by rolling dry polystyrene sticks (8 mm diameter by 102 mm length) on the leaf canopies. The sticks coated with the mucilage were individually placed in 15-mL Corning tubes and centrifuged 2050×*g* for 5 min. The precipitated mucilage, typically 15 to 30 μL per tube/stick, was collected with a Pasteur pipette and pooled into a 50-mL Corning tube, and kept frozen at −20 °C. The mucilage was collected three or four times a week. When a combined total of 10 mL mucilage was obtained, the pooled mucilage was defrosted, mixed with water (40 mL) and left overnight at 4 °C to yield viscous *but not elastic* solution. This was filtered through a 20-μm porosity polythene frit as above. The filtered mucilage solution was transferred to a 100-mL Duran bottle and freeze-dried to yield 200.8 mg of solid, which was stored at room temperature in dry condition. This material was re-dissolved in water (20 mL) at 80 °C, and treated with 80 mL iPrOH as above after cooling to the room temperature. The yields of iPrOH-soluble and iPrOH-insoluble fractions were 78.4 and 122.5 mg, respectively.

**Fig. 2:**
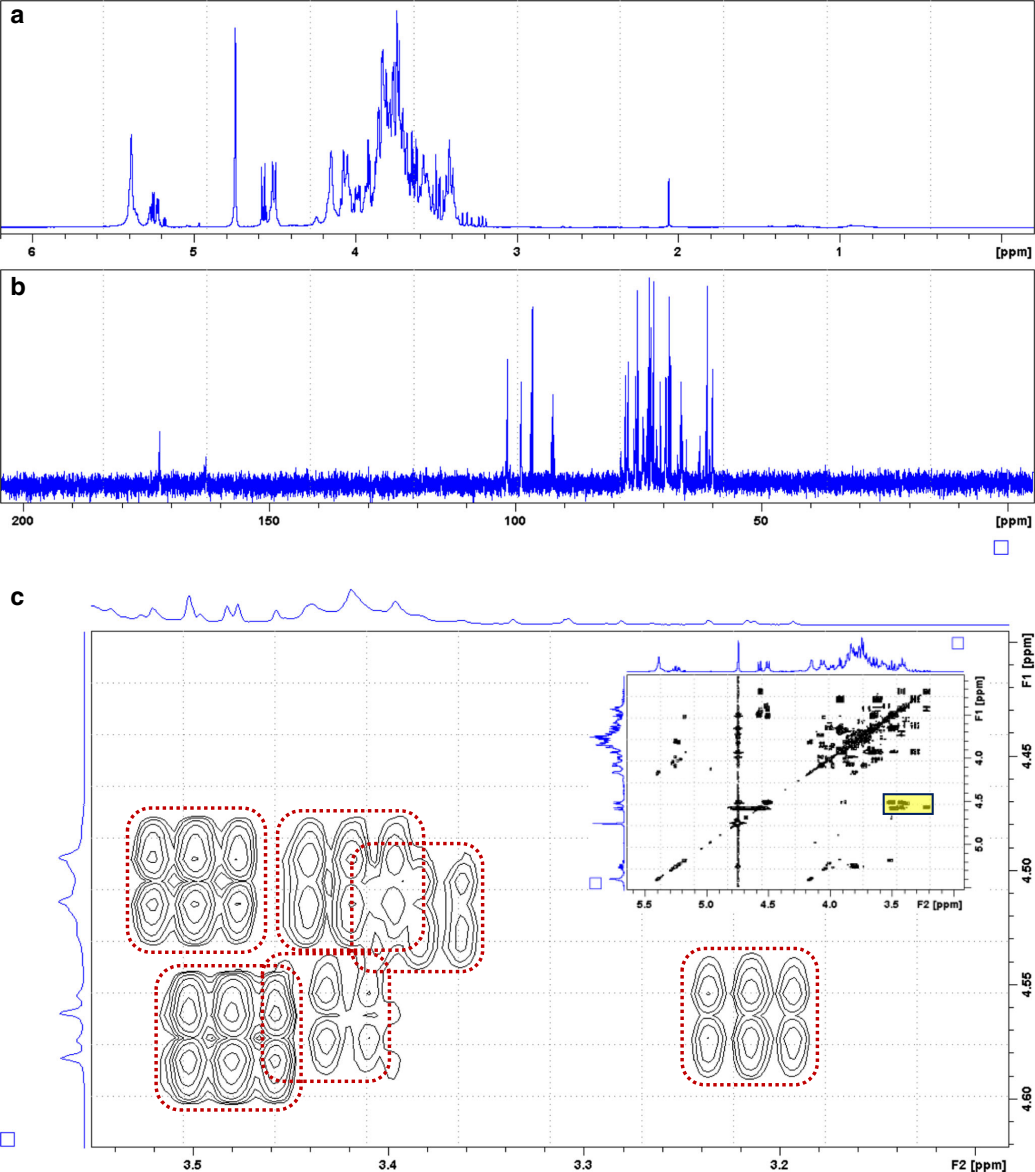
NMR spectra (D_2_O, 30 °C) of acid hydrolysed polysaccharide component (trifluoroacetic acid, 2 M, 100 °C, 4 h). **a** Proton spectrum with HDO presaturation. **b** Carbon-13 spectrum (data acquisition 10 s). **c** COSY spectrum, expansion of the region of β-configured carbohydrates C-1[*F*_1_]–C-2[*F*_2_] cross-correlations. Inset: full correlation contour plot

The iPrOH-soluble fraction was used for acquisition of full NMR dataset (online resource Fig. S1–S5). A portion of the iPrOH-insoluble material (57.1 mg) was hydrolysed with 2 M trifluoroacetic acid (TFA) at 100 °C for 4 h (Rost and Schauer 1977; Redgwell 1983). The resulting hydrolysate was dried first on a heat block (50 °C) and at a high vacuum (approx. 0.1 mBar) overnight before an NMR analysis (1D ^1^H, 1D ^13^C and COSY; Fig. 2).

### Mucilages of *Abelmoschus*, *Actinidia*, *Aloe*, *Lupinus* and *Zea*

Historically well-studied mucilage samples were obtained for comparative analysis as follows: *Abelmoschus esculentus* (L.) Moench. (okra, lady’s fingers; syn. *Hibiscus esculentus* L.; Malvaceae): fresh fruits were obtained from local grocer. The remains of the flower at the top of the fruits were cut off with a knife, and the seed cavities were rinsed with D_2_O, applying a gentle rolling motion while keeping the fruit upright. The mucilage solution was recovered by a gentle centrifugal force, before centrifugation further at 12,000×*g* to remove solid particulates. *Actinidia deliciosa* (A.Chev.) C.F.Liang & A.R.Ferguson (kiwifruit cv. ‘Hayward’, as used by Regdwell (1983) and currently classified): the seeds were retrieved from commercially available fruits and grown initially on seed compost then on soil mix, to the height of approx. 60 cm on a cane (approx. 20-month old). The mucilage extraction method closely followed that applied to bulk pith by Redgwell (1983), except that the stem pith fragments were immersed in D_2_O and that the debris were removed by centrifugation ready for NMR analysis. The viscosity of the solution indicated the successful extraction of the mucilage. *Aloe vera* (L.) Burm.f. (Xanthorrhoeaceae) was a common ornamental variety, grown in a 9-cm pot. Similarly to *Actinidia*, the mesophyll mucilage was directly dispersed into D_2_O, followed by centrifugation. *Lupinus polyphyllus* Lindl. (lupin, cv ‘Russell’; Fabaceae) and *Zea mays* L. (sweetcorn, cv ‘Incredible’; Poaceae): The seeds were obtained from Johnsons (Suffolk, UK), and germinated using the methods described by Read and Gregory (1997) on 9-cm diameter Petri dishes, seven seeds per dish. Film or droplets of mucilage were observed on the surfaces of root tips that were not in contact with the wet paper in the Petri dishes (Read and Gregory 1997). However, they were in such small quantity and highly viscous that they could not be collected by pipetting or with a capillary tube. Thus, the roots were immersed and the mucilages washed into two Eppendorf tubes (per species) each containing 500 μL D_2_O. The mucilage solutions were combined species-wise and centrifuged, obtaining approx. 1 mL viscous solutions from each of the species.

## Results

### Direct NMR profiling

The initial proton NMR analysis (at 30 °C) of the mucilage collected by centrifuge exhibited, as expected, a large dominant signal of H_2_O/HDO. Therefore, all subsequent profiling was conducted with HDO presaturation applied (typically at 4.70–4.71 ppm). Although the H_2_O/HDO signals could not be eliminated completely, the signals of solute origin could be observed (Fig. 1a). Consistent with the viscosity and polysaccharide structures, both the anomeric protons (5.5–5.1 ppm) and ring protons (4.3–3.2 ppm) appeared as broad bands rather than sharp signals. However, the most striking feature of the spectrum was the four sets of sharp signals at 4.05 (*t*, coupling constant 2.9 Hz), 3.61 (*dd*, 10.0 and 9.3 Hz), 3.52 (*dd*, 9.9 and 2.9 Hz) and 3.27 (*t*, 9.3 Hz) ppm. The sharpness of the signals indicated the absence of paramagnetic elements and ions in the sample solution, as well as that the molecular motion of this component was not restricted. With the same reasoning, the mucilage was devoid of free carbohydrate monomers and oligomers of aldose or ketose, since no anomeric proton with well-defined coupling constants could be observed in 5.1 ppm (α, *J* = 1–4 Hz) or 4.6 ppm (β, *J* = 6–8 Hz) regions (Agrawal 1992). A series of broad signals could also be observed between 2.3 and 0.8 ppm region, among which the one at 1.29 ppm exhibited a significant intensity.

Raising the sample temperature to 60 °C did not improve the resolution of the signals attributable to polysaccharides. The only differences were the chemical shift values of some signals (data not shown). The sample was kept at this temperature for about an hour; a re-taken proton spectrum at 30 °C was found to be identical to the very original one, indicating no degradation. Subsequent analyses were therefore all conducted at 30 °C.

The mucilage samples collected exclusively from leaves with red tentacles and from those with white tentacles both exhibited the polysaccharides and the same four sets of sharp signals (Fig. 1a, b). There were several low-intensity signals unique to each of the colour forms, but these were not analysed further. Both of the polysaccharide and the yet unidentified compound(s), giving the four sharp signals, were observed also in the two mucilage samples (D1, D2) regenerated by the detached leaves (Fig. 1c, d).

### Identification of the ‘free’ compound

The separation of the compound that gave the characteristic four signals could be achieved conveniently by precipitating the polysaccharide with isopropanol (iPrOH). The supernatant fraction (iPrOH-H_2_O, 4:1, *v*/*v*) was practically pure sample of the compound of interest, as far as the readily D_2_O-soluble part (in 600 μL, room temperature) was concerned (Fig. 1e, S1). The small amount of insoluble material was not studied further.

In the ^13^C NMR spectrum, only four signals could be detected, and they were all *sp* ^3^-carbinols as indicated by the chemical shifts of 74.9, 73.0, 72.8 and 71.7 ppm (Fig. S2). The signals at 73.0 and 71.7 ppm were of double intensities compared to the other two (FID acquisition time 10 s), therefore a six-carbon compound was suggested. The multiplicity-edited HSQC spectrum revealed that all carbons bore one proton each, consistent also with the integration of the proton signals, where the triplet at 3.61 ppm and double-doublet at 3.52 ppm represented two protons each. Thus, a six-membered ring with an element of symmetricity was suggested. The examination of the coupling constants of the carbinol protons allowed the orientation of the hydroxyl groups to be deduced as [*eq*-, *ax*-, *eq*-, *eq*-, *eq*-, *eq*]-sequence, leading to the identification of *myo*-inositol (MI). The COSY, HSQC and HMBC spectra were also fully consistent with this identification (Fig. S3–S5). An authentic sample of *myo*-inositol gave identical proton and ^13^C NMR spectra (Fig. S1, S2).

### Polysaccharide fractions

When the iPrOH-insoluble part was dried under high vacuum, it turned to a very hard pellet (cf. Rost and Schauer 1977), which could only be swollen even in copious amount of water for days. It formed a lumpy mass, but did not dissolve. When heated to 80 °C, it turned to viscous gel solution but *without elasticity*. After cooling to room temperature, the solution remained so as relatively free-flowing gel unlike that of, for example, agar or agarose. The solution in D_2_O exhibited an identical proton NMR spectrum with that of raw mucilage except that it contained only a trace of MI (Fig. 1f). The resolution could not be improved after repeated dilution in D_2_O, consistent with the very high molecular weight; MW of greater than 2 × 10^6^ has been proposed by Rost and Schauer (1977). At higher dilution, the signals eventually diminished to below the limit of detection, therefore no further insight could be obtained.

The spectra of TFA-hydrolysed polysaccharide showed severe overlapping of signals but could be recognised readily as those of carbohydrate monomers. Thus, two sets of anomeric proton clusters corresponding to α-configurations (*δ*_H_ ~ 5.2 ppm) and β-configurations (*δ*_H_ ~ 4.5 ppm) could be observed in the ^1^H spectrum (Agrawal 1992) (Fig. 2a). In the carbon-13 spectrum, a carbonyl carbon could be detected at *δ*_C_ 172.3 ppm, corresponding to the C-6 position of an uronic acid (Agrawal 1992) (Fig. 2b). The β-anomeric region of the COSY spectrum showed six distinct monomer units, in contrast to the five that were previously identified (l-Ara, d-Xyl, d-Gal, d-Man, d-GlcA; Gowda et al. 1983) (Fig. 2c). Because of the severe overlapping of the signals even in the dispersed 2D spectra, exacerbated by the spontaneous configurational changes between the α- and β-forms of the individual monosaccharides, it was not possible to trace the spin systems beyond the C-1–C-2 positions of the component carbohydrate units in this work.

The retentate polysaccharide fraction from the dialysis procedure gave a fibrous solid (Rost and Schauer 1977). It could only be dissolved properly at high temperature in D_2_O at 80 °C. Unexpectedly, the solution was viscous *and elastic* when cooled down. Both of the iPrOH-insoluble and dialysis retentate polysaccharides retained some degree of adhesiveness onto various surfaces as observed under microscope; this observation will be discussed in more detail below. The 1D and 2D NMR spectra of the retentate were broadly identical to those of iPrOH-insoluble fraction, except that the dialysis had removed MI and other small MW components completely (Fig. 1f, g). A detailed comparison of the datasets revealed noteworthy common features in the polysaccharide samples prepared in different manners. First, three strong HSQC cross-correlations were detected at *δ*_C_ 30.5/*δ*_H_ 1.3, *δ*_C_ 14.6/*δ*_H_ 0.93 and *δ*_C_ 54.2/*δ*_H_ 3.85, corresponding to methylene (–CH_2_–), terminal alkyl-methyl (–CH_3_) and methoxyl (–OCH_3_) groups, respectively (Fig. S6). The widths and the integrations of the corresponding proton signals in the 1D spectra suggested that the methylene group(s) formed parts of alkyl chain. Second, the HMBC spectra (Fig. S7) showed strong correlations between *δ*_C_ 171.0 and *δ*_H_ 3.85, which could only be interpreted as a methoxycarbonyl partial structure **C**O–O–C**H**_**3**_, consisting of the methoxyl group mentioned above. No other proton could be detected to correlate with the carbonyl carbon, however. This methoxyl group was not observed in the HSQC spectrum of the TFA-hydrolysed material (data not shown).

### Comparison against the mucilage of *Actinidia*, *Abelmoschus*, *Aloe*, *Lupinus* and *Zea*

None of the other mucilage samples from different species contained detectable amount of MI (Fig. S8). They, however, all contained different low MW components unique to each of the species. Since the signals were sharp, it could be deduced that those compounds were unbound to the polysaccharide components. Whilst the fine-structures of the signals of the polysaccharides were severely obscured due to the viscosity and MW, they also exhibited overall shape unique to the species. However, none of them were superimposable to the polysaccharide component of *Drosera* mucilage (Fig. S9).

## Discussion

### *myo*-Inositol

From the spectroscopic analyses and the dry weights of mucilage as a whole, its separated components and the producing leaves, it may be deduced that (1) the mucilage is approximately 2% solution of mostly organic substances; (2) the mucilage represents approximately 4.3% of the solid mass (loosely photosynthate) of the leaf, including the green, presumably photosynthesis-capable, petiole; (3) approximately 65% of the solute by weight is polysaccharide consisting of six carbohydrate monomer units; and (4) *myo*-inositol (MI) represents a significant proportion of the non-polysaccharide organic components.

It appears that MI was overlooked because of the commonly applied techniques in the chemical analysis of mucilage, involving dialysis followed by acid (or enzymatic) hydrolysis and detection of the component carbohydrate units (Rost and Schauer 1977; Redgwell 1983). A dialysis experiment on the freshly harvested mucilage not only removed MI completely but also other co-occurring low MW components (Fig. 1g). The absence of the target reaction site for reagents used for aldoses and ketoses and a chromophore were clearly the additional factors for confounding its detection (Loewus 1990). Considering also that the plants used in 1977 and current work were both grown in glasshouses at well comparable latitudes in the Northern hemisphere, it is unlikely that the growth conditions or the associated stresses were significant cause of the high MI concentration.

The unusually high concentration of MI deserves a consideration, especially in relation with the plants’ habitat. MI is a primary metabolite, biosynthesised from glucose in two steps and plays pivotal roles in the biochemistry of plants (Loewus and Murthy 2000). It is also the direct precursor of glucuronic acid, a component of the main mucilage polysaccharide, although the conversion of MI to GlcA then incorporation into the polysaccharide in situ in the exuded mucilage is unlikely. On the other hand, the ratio of approximately one molecule of MI for every two carbohydrate monomer units of the polysaccharide suggest that the MI might act as a cross-linker (hydrogellator) between the polysaccharide strands, presumably through the formation of a hydrogen bond-network between the hydroxyl groups (see Du et al. (2015) for comprehensive review of hydrogels and hydrogellators; sections 4.1.4 and 4.1.11 in this paper are the most relevant). The sharp proton NMR signals are consistent with this view, in that the molecular motion of MI is not restricted in the solution state. The retention of the elasticity in the exceedingly diluted mucilage could also be explained by this mechanism, because of the six hydroxyl groups per molecule of MI acting as a hub. Additionally, MI may function as an osmoticum to draw water from the gland and retain it in the hydrogel; the tentacles are directly connected to the vascular system to facilitate this process (Williams and Pickard 1974; Adlassnig et al. 2010). An analogous mechanism for MI has been proposed in the case of the rapidly developing fruits of *Actinidia arguta* (Klages et al. 1998). This would be particularly relevant in order to counter the evaporation from vast surface area per volume of the droplets, which are generally fully exposed to the sunlight in the plants’ natural habitat. The complete drying of the mucilage by artificial means under vacuum was earlier noted to lead to the loss of the viscosity (Rost and Schauer 1977). Interestingly, the shrinkage of the glandular heads has been observed after the secretion of the mucilage from the stalked glands (Naidoo and Heneidak 2013), suggesting strongly that the transfer of the cell contents to the mucilage was by suction from outside rather than by positive pressure from the cells to the exterior matrix.

The biosynthesis of MI is relatively ‘cheap’, being generated stoichiometrically from one mole of glucose only by isomerisation (Loewus and Murthy 2000). As demonstrated by the excised leaves, the biosynthesis of MI can be completed right in the photosynthetic organ alone. On the other hand, being exudate it is an expensive (or expendable) investment since it may be lost easily and repeatedly in rainfall during the lifetime of the producing leaves. The complete loss of MI in the dialysis experiment against pure water without the use of chaotropic agents suggests that the MI can be separated easily from the polysaccharide components. Therefore, the biosynthesis of MI (as well as that of the polysaccharide components) would represent a significant investment for a potential return which is, however, not guaranteed. The fate of the lost MI is therefore an interesting line of query, to which a particularly tempting hypothesis is proposed here that MI may selectively enrich or activate the population of nitrogen-fixing bacteria (NFB) in the soil. MI is a known signal molecule for NFB, and the MI-catabolising activity is strongly associated with biological N-fixation (Fry et al. 2001; Sullivan et al. 2013; Yoshida et al. 2006). Waterlogged habitats such as bogs and fens are especially suitable for this to take place, since they provide the anaerobic environment for dinitrogenase of the NFB that are associated with submerged parts of plants (Doroshenko et al. 2007; Leppänen et al. 2015). There have been observations that some carnivorous plants are modifying their local bacterial compositions with unknown mechanisms (Aira et al. 2015; Sickel et al. 2016). Indeed, putative diazotrophic bacteria have been reported to be enriched in the rhizosphere and in the root tissues of a related species *Drosera villosa*, despite the presumably hostile acidic environment for the bacteria (Albino et al. 2006). Access to inorganic N through root is advantageous, since the N is immediately useable to incorporate into metabolic grids without further digestion (Kraiser et al. 2011; Millett et al. 2015). Potentially, N-fixing bacteria have also been found on the pitcher of *Sarracenia minor* (Sarraceniaceae) and in the acidic fluids (Siragusa et al. 2007). The fluid has minimal interface with stagnant air in the pitcher, conceivably providing an anaerobic condition. In the case of *Sarracenia purpurea*, the associated NFB in the pitchers were potentially capable of meeting the entire N-requirement of the plant (Prankevicius and Cameron 1991). Its sister taxa *Darlingtonia* and majorities of *Heliamphora* in the same family Sarraceniaceae lack their own digestive enzymes and so dependent on bacteria for N acquisition (Chase et al. 2009). The presence of NFB has also been confirmed inside the traps of the aquatic carnivorous plants of *Utricularia* (Lentibulariaceae) (Sirová et al. 2014), although those NFB may be bycatch in the same way inanimate microalgae and pollens are sucked into the traps (Koller-Peroutka et al. 2015). Overall, the bacterial associates may be underappreciated source of N beside animal preys, their predators at higher trophic levels, and atmospheric deposition (Millett et al. 2015; Pavlovič et al. 2016).

There are two obvious caveats to the NFB-enrichment hypothesis, one of which is that the released MI would be dispersed well beyond the producing plants in the wetland, in contrast to the rhizodeposition that manipulate the functional microbial community structures only at the rhizosphere region close to the root surfaces (Chaparro et al. 2012; Paterson et al. 2007). The other is that whilst MI catabolism is associated with biological N-fixation, it is by no means unique to NFB. The role of root-associated fungi cannot be discounted due to the lack of evidence, although the available information seems to suggest their contribution to be minimal as the suppliers of either N or P. Thus, Fuchs and Haselwandter (2004) reported that the roots of *Drosera intermedia* were colonised only occasionally by arbuscular mycorrhizal fungi (AMF) (1–7% root length), and not by endophytic fungi at all. Phosphorus is, at any rate, a limited resource in bogs (Eber 1982). These considerations supports further the hypothesis that the acquisition of phosphorus rather than nitrogen is the principle purpose of carnivory (Ellison 2006).

MI is otherwise known to possess sweetness and act as a stimulant of feeding of a number of insect species, especially for the larvae of herbivore insects (Schoonhoven and van Loon 2002). However, this is certainly irrelevant for attracting and retaining of the preys, not only because MI is non-volatile but also the landing of flying preys may be merely ‘accidental’ without sensory cue (discussed by Jürgens et al. 2009 and by Potts and Krupa 2016). Therefore, MI is unlikely to be a cause of, if ever, different attractiveness behind the colour phenotypes, although the relative concentrations of MI to polysaccharide was greater in the white form than red form of tentacles (Fig. 1a, b). The general consensus is inclined toward irrelevance of colours between red and white forms, based on the bichromic vision systems of most insect preys as well as from experimental grounds (Bennett and Ellison 2009; Foot et al. 2014; Pavlovič et al. 2014; Potts and Krupa 2016). Furthermore, MI and polysaccharides are both transparent to UV light, implying also that there is no fluorescent emission at any longer wavelengths (e.g. in ‘visible’ 400–800 nm) range. The NMR spectrum of the raw mucilage showed no aromatic or alkenic proton in the *δ*_H_ 8.2–5.5 ppm (Fig. S9). This is in line with the insignificant UV reflectance patterns of the *Drosera* mucilage drops (Joel et al. 1985).

### Viscosity, elasticity and adhesiveness

The discrepancies between the reported properties of mucilages from *Actinidia* and *Drosera* were the primary driver of current study. It appears that the NMR spectra of polysaccharide components in these mucilages are indeed different (Fig. S8b and S8f) both qualitatively and in concentration relative to other components. MI was not detected in *Actinidia* mucilage extract, even though the diffusate from the pith or the other parts of the stem was conceivably present in the sample. Only that from the root tips of *Zea mays* had a marginal elasticity (Read and Gregory 1997) and adhesiveness compared to *Drosera*’s; but again, it did not contain a detectable amount of MI. The dialysed *Drosera* mucilage retained the elasticity and adhesiveness, therefore it is unlikely that MI contributes to them at least directly. Neither the iPrOH-insoluble fraction nor the solution of authentic MI exhibited the elasticity; re-mixing of the components at any proportions again failed to restore the original property. Therefore, there seems as yet unidentified factor beyond a simple composition analysis would suggest. It is likely to be based on a structural organisation at higher order, fabricated at the time of mucilage exudation where a strand of mucilage could be observed (Williams and Pickard 1974; Huang et al. 2015). The intricate organisation may be irreversibly disrupted by chemical (e.g. pH) or physical (e.g. temperature, including freezing) factors, as well as methods of drying (Rost and Schauer 1977, and this work). In the natural environment, however, the mucilage needs only to maintain its visco-elasticity and adhesiveness seasonally and within the temperature range when potential preys are around.

The mucilage of *Drosera* has a remarkable adhesiveness, which is an important property for the purpose of trapping and retaining the preys. Indeed, the mucilage binds to different parts of insects’ bodies such as waxy exoskeletons, setae and the wings of diptera and lepidoptera. The wings have microscopic water-repellent features in the forms of setae and porous microstructures (Darmanin and Guittard 2015). Upon contact, the mucilage spreads and adheres to these structures and cuticles in between (unpublished observation). Furthermore, the mucilage instantly adhere to the nature’s well-known anti-adhesive leaf surfaces of *Nelumbo nucifera* (the ‘Lotus-effects’, Barthlott and Neinhuis 1997), *Sedum* sp., *Macleaya cordata*, *Brassica oleracea* and *Ginkgo biloba* (unpublished observation), all, coincidentally, plant origins.^1^ These superhydrophobic plant surfaces are based on waxy substances in species-specific intricate microstructures on the epidermis (Neinhuis and Barthlott 1997). Therefore, it could be envisaged that the mucilage binds to biological materials of a range of surface properties, from pollen grains which often have mucilage coatings of their own (‘pollenkitt’; Hesse 2010), to human hair as Darwin himself experimented (Chase et al. 2009). The precise mechanism for this adhesiveness remains unknown. Huang et al. (2015) have measured the contact angle of the intact *Drosera* mucilage, showing that it had substantially lower surface tension than water. The presence of a surfactant is therefore a possibility, but this is not entirely consistent with how the mucilage droplets form and stay at the tip of the tentacle, instead of flowing on the stalk toward the leaf blade (Adlassnig et al. 2010; Naidoo and Heneidak 2013). The 2D NMR spectra of the polysaccharide fractions (HSQC and HMBC, Fig. S6, S7) showed the presence of lipophilic moieties such as carboxylic acid methyl ester and alkyl groups, but the exact structure of the latter, or how they form the part of the polysaccharide component, could not be clarified through a direct NMR analysis. These functional groups were presumably lost following the acid hydrolysis with TFA, as methanol from the methyl ester and as volatile short chain alcohols or acids.

### Inference on inorganic components

The employed NMR spectroscopic techniques were suitable only for organic components. Any mineral components in the mucilage were therefore undetected in this work. However, the reported mineral compositions of the *Drosera* mucilage are, in the author’s opinion, questionable. Thus, the occurrence and/or concentrations of Ca^2+^ (22 mM, = 880 mg Ca^2+^/L = 0.044 N) and Mg^2+^ (19 mM, = 456 mg Mg^2+^/L = 0.038 N) might be erroneous (see Rost and Schauer 1977; Zhang et al. 2010). The typical habitats of *Drosera* spp. are bogs, where these minerals are practically absent or at low ppm concentrations (Adamec 1997; Eber 1982; Jones et al. 2016). In fact, Ca^2+^ is often detrimental to the calcifuge species inhabiting in ombrotrophic wetlands (Adlassnig et al. 2005; Jones et al. 2016). In comparison, the UK classification of ‘very hard water’ is 300 mg/L CaCO_3_ or above, equivalent to mere 120 mg/L Ca^2+^. The reported concentrations of the divalent cations are therefore well excess of neutralising the acidic mucilage polysaccharide, even if 50% of it is assumed to be glucuronic acid in the free acid form (~0.038 N).^2^ The visco-elasticity and the adhesiveness of the regenerated mucilages from detached leaves were indistinguishable from those accumulated by the attached leaves. It implies that the functional mucilage could be regenerated from water, natural daylight and root- or soil-derived resources that were stored and available in the leaves; the organic mucilage components were all biosynthesised in the leaves but not translocated from elsewhere. Increased production of stickier mucilage under higher solar radiation has been noted previously for *Drosera rotundifolia* (Thorén et al. 2003). Therefore, the availability of water and light, and in turn the photosynthate, are the primary determinant of its production; the increased production of MI at the same time would protect the mucilage’s function as discussed above. Alternatively, if the concentrations are indeed as originally reported, it may be the result of active disposal of unwanted but passively absorbed minerals from the prey and to the lesser extent from the soil (Adamec 1997; Pavlovič 2014). In the case of the aquatic carnivorous plant *Aldrovanda* (Droseraceae), Ca and Mg (as well as Na and K) were considered to be lost altogether with the senescing (and dying) whorls without being remobilised (Adamec 1997).

## Concluding remarks

The mucilage of *Drosera* spp. is a fascinating material: its visco-elasticity and adhesiveness remain functional even after prolonged exposure to sunlight typical for the habitats of the Droseraceae. It is considered unsupportive of microbial growth (Adlassnig et al. 2010), despite constantly moist and fully exposed to the environment. Upon prey capture, it does not interfere with the subsequent digestion. The adhesiveness on the preys’ water-repellent body parts discussed above would suggest a coating function for maximising the areas of contact for the digestive enzymes to act upon. That some captured prey may escape after some time may indicate that the mucilage is free from a component that are immediately toxic to the potential prey (Gaume and Forterre 2007). It is therefore no surprise that the mucilage attracted attention of material scientists.

Apart from the specific functions for *Drosera*, mucilage occurs ubiquitously in the plant kingdom, playing roles as forms of storage for carbohydrates and water as hydrogels, attracting and sticking to the pollinators and aiding seed dispersal by adhering to transporting animals. They also play parts in resisting grazing herbivores, and presenting mechanical barrier to fungal and bacterial infection especially after injury. Beside the direct implications in the producing plants’ successful life cycles, they have been used as food sources and for medicinal purposes (often as dressings; Morton 1990), as well as in numerous artefacts for example ink, paints cosmetics, glue substitute and as lubricants. Mucilages are typically characterised by physical properties such as solubility in water, rheology and adhesive properties, and chemically defined (together with gums and hydrocolloids) as non-starch polysaccharides (Cummings and Stephen 2007). However, their chemical compositions are probably inadequately characterised beyond the polysaccharide components. In all likelihood, the description of polysaccharide components is also incomplete especially if acid hydrolysis was used. It may be apposite to reconsider some of the techniques used routinely in the past. All or some of the properties mentioned above are potentially excellent model for developing a functional material; however, a rigorous analysis is required before we may fully appreciate its true natural composition, organisation and the mechanism of actions (Payne 2007). Only then would it be possible to manipulate the properties for specific applications (Fratzl 2007).

## Electronic supplementary material

ESM 1 (PDF 387 kb).

## Footnotes

1 Water-based adhesives such as PVA glue roll off some of these superhydrophobic surfaces. Neat kitchen detergents will begin to make adhesive contact after a short while if balanced horizontally.

2 In the backbone of the proposed co-polymer (–GlcA–Man–)_n_, these carbohydrate units are represented by C_6_H_8_O_6_ (‘MW’ 176) and C_6_H_10_O_5_ (‘MW’ 162) respectively, because of the loss of one mole of water at each linkage point. Assuming that the MW is sufficiently large, and that approximately 65% of the total mucilage dry matter (2% *w*/*v*) is the polysaccharide, GlcA represents 20 × 0.65 × 176/(176 + 162) = 6.769 g/L, which is equivalent to 6.769 / 176 = 0.0384 mol/L (= 0.0384 N). This value would drop to 0.0253 N when based on the reported monosaccharide composition (Gowda et al. 1983).

## References

[CR1] Adamec L (1997). Mineral nutrition of carnivorous plants: a review. Bot Rev.

[CR2] Adlassnig W, Lendl T, Peroutka M, Lang I, von Byern J, Grunwald I (2010). Deadly glue – adhesive traps of carnivorous plants. Biological adhesive systems: from nature to technical and medical application.

[CR3] Adlassnig W, Peroutka M, Lambers H, Lichtscheidl IK (2005). The roots of carnivorous plants. Plant Soil.

[CR4] Agrawal PK (1992). NMR spectroscopy in the structural elucidation of oligosaccharides and glycosides. Phytochemistry.

[CR5] Aira M, Bybee S, Domínguez J (2015). Carnivory does not change the rhizosphere bacterial community of the plant *Drosera intermedia*. Appl Soil Ecol.

[CR6] Albino U, Saridakis DP, Ferreira MC, Hungria M, Vinuesa P, Andrade G (2006). High diversity of diazotrophic bacteria associated with the carnivorous plant *Drosera villosa* Var. *villosa* growing in oligotrophic habitats in Brazil. Plant Soil.

[CR7] Aspinall GO, Puvanesarajah V, Reuter G, Schauer R (1984). Selective cleavage of *β*-D-glucopyranosiduronic acid linkages in methylated polysaccharide acids from *Drosera* species. Carbohydr Res.

[CR8] Barthlott W, Neinhuis C (1997). Purity of the sacred lotus, or escape from contamination in biological surfaces. Planta.

[CR9] Bennett KF, Ellison AM (2009). Nectar, not colour, may lure insects to their death. Biol Lett.

[CR10] Chaparro JM, Sheflin AM, Manter DK, Vivanco JM (2012). Manipulating the soil microbiome to increase soil health and plant fertility. Biol Fertil Soils.

[CR11] Chase MW, Christenhusz MJM, Sanders D, Fay MF (2009). Murderous plants: Victorian gothic, Darwin and modern insights into vegetable carnivory. Bot J Linn Soc.

[CR12] Cummings JH, Stephen AM (2007). Carbohydrate terminology and classification. Eur J Clin Nutr.

[CR13] Darmanin T, Guittard F (2015). Superhydrophobic and superoleophobic properties in nature. Mat Today.

[CR14] Doroshenko EV, Boulygina ES, Spiridonova EM, Tourova TP, Kravchenko IK (2007). Isolation and characterization of nitrogen-fixing bacteria of the genus *Azospirillum* from the soil of a *Sphagnum* peat bog. Microbiology.

[CR15] Du X, Zhou J, Shi J, Xu B (2015). Supramolecular hydrogelators and hydrogels: from soft matter to molecular biomaterials. Chem Rev.

[CR16] Eber W (1982). The ecology of bogs and bog plants. J Life Sci, R Dublin Soc.

[CR17] Egan PA, van der Kooy F (2013). Phytochemistry of the carnivorous sundew genus *Drosera* (Droseraceae) – future perspectives and ethnopharmacological relevance. Chem Biodivers.

[CR18] Ellison AM (2006). Nutrient limitation and stoichiometry of carnivorous plants. Plant Biol.

[CR19] Foot G, Rice SP, Millett J (2014). Red trap colour of the carnivorous plant *Drosera rotundifolia* does not serve a prey attraction or camouflage function. Biol Lett.

[CR20] Fratzl P (2007). Biomimetic materials research: what can we really learn from nature’s structural materials?. J R Soc Interface.

[CR21] Fry J, Wood M, Poole PS (2001). Investigation of *myo*-inositol catabolism in *Rhizobium leguminosarum* bv. *viciae* and its effect on nodulation competitiveness. Mol Plant-Microbe Interact.

[CR22] Fuchs B, Haselwandter K (2004). Red list plants: colonization by arbuscular mycorrhizal fungi and dark septate endophytes. Mycorrhiza.

[CR23] Gaume L, Forterre Y (2007). A viscoelastic deadly fluid in carnivorous pitcher plants. PLoS One.

[CR24] Gottlieb HE, Kotlyar V, Nudelman A (1997). NMR chemical shifts of common laboratory solvents as trace impurities. J Org Chem.

[CR25] Gowda DC, Reuter G, Schauer R (1982). Structural features of an acidic polysaccharide from the mucin of *Drosera binata*. Phytochemistry.

[CR26] Gowda DC, Reuter G, Schauer R (1983). Structural studies of an acidic polysaccharide from the mucin secreted by *Drosera capensis*. Carbohydr Res.

[CR27] Hesse M, von Byern J, Grunwald I (2010). Bonding single pollen grains together: how and why?. Biological adhesive systems: from nature to technical and medical application.

[CR28] Huang Y, Wang Y, Sun L, Agrawal R, Zhang M (2015). Sundew adhesive: a naturally occurring hydrogel. J R Soc Interface.

[CR29] Joel DM, Juniper BE, Dafni A (1985). Ultraviolet patterns in the traps of carnivorous plants. New Phytol.

[CR30] Jones JMC, Massicotte HB, Fredeen AL (2016). Calcium and pH co-restrict abundance of *Drosera rotundifolia* (Droseraceae) in a *Sphagnum* bog in central British Columbia. Botany.

[CR31] Jürgens A, El-Sayed AM, Suckling DM (2009). Do carnivorous plants use volatiles for attracting prey insects?. Funct Ecol.

[CR32] Klages K, Donnison H, Boldingh H, MacRae E (1998). *myo*-inositol is the major sugar in *Actinidia arguta* during early fruit development. Aust J Plant Physiol.

[CR33] Koller-Peroutka M, Lendl T, Watzka M, Adlassnig W (2015). Capture of algae promotes growth and propagation in aquatic *Utricularia*. Ann Bot.

[CR34] Kraiser T, Gras DE, Gutiérrez AG, González B, Gutiérrez RA (2011). A holistic view of nitrogen acquisition in plants. J Exp Bot.

[CR35] Król E, Płachno BJ, Adamec L, Stolarz M, Dziubińska H, Trębacz K (2012). Quite a few reasons for calling carnivores ‘the most wonderful plants in the world’. Ann Bot.

[CR36] Lenaghan SC, Serpersu K, Xia L, He W, Zhang M (2011). A naturally occurring nanomaterial from the sundew (*Drosera*) for tissue engineering. Bioinsp Biomim.

[CR37] Leppänen SM, Rissanen AJ, Tiirola M (2015). Nitrogen fixation in *Sphagnum* mosses is affected by moss species and water table level. Plant Soil.

[CR38] Loewus FA, Dey PM (1990). Cyclitols. Methods in plant biochemistry.

[CR39] Loewus FA, Murthy PPN (2000). *myo*-inositol metabolism in plants. Plant Sci.

[CR40] Millett J, Foot GW, Svensson BM (2015). Nitrogen deposition and prey nitrogen uptake control the nutrition of the carnivorous plant *Drosera rotundifolia*. Sci Total Environ.

[CR41] Morton JF (1990). Mucilaginous plants and their uses in medicine. J Ethnopharmacol.

[CR42] Naidoo Y, Heneidak S (2013). Morphological investigation of glandular hairs on *Drosera capensis* leaves with an ultrastructural study of the sessile glands. Botany.

[CR43] Neinhuis C, Barthlott W (1997). Characterization and distribution of water-repellent, self-cleaning plant surfaces. Ann Bot.

[CR44] Paterson E, Gebbing T, Abel C, Sim A, Telfer G (2007). Rhizodeposition shapes rhizosphere microbial community structure in organic soil. New Phytol.

[CR45] Pavlovič A, Krausko M, Adamec L (2016). A carnivorous sundew plant prefers protein over chitin as a source of nitrogen from its traps. Plant Physiol Biochem.

[CR46] Pavlovič A, Krausko M, Libiaková M, Adamec L (2014). Feeding on prey increases photosynthetic efficiency in the carnivorous sundew *Drosera capensis*. Ann Bot.

[CR47] Payne GF (2007). Biopolymer-based materials: the nanoscale components and their hierarchical assembly. Curr Opin Chem Biol.

[CR48] Potts L, Krupa JJ (2016). Does the dwarf sundew (*Drosera brevifolia*) attract prey?. Am Midl Nat.

[CR49] Prankevicius AB, Cameron DM (1991). Bacterial dinitrogen fixation in the leaf of the northern pitcher plant (*Sarracenia purpurea*). Can J Bot.

[CR50] Read DB, Gregory PJ (1997). Surface tension and viscosity of axenic maize and lupin root mucilages. New Phytol.

[CR51] Redgwell RJ (1983). Composition of *Actinidia* mucilage. Phytochemistry.

[CR52] Rost K, Schauer R (1977). Physical and chemical properties of the mucin secreted by *Drosera capensis*. Phytochemistry.

[CR53] Schlauer J, Nerz J, Rischer H (2005). Carnivorous plant chemistry. Acta Bot Gallica.

[CR54] Schoonhoven LM, van Loon JJA (2002). An inventory of taste in caterpillars: each species its own key. Acta Zool Acad Sci Hung.

[CR55] Sickel W, Grafe TU, Meuche I, Steffan-Dewenter I, Keller A (2016). Bacterial diversity and community structure in two Bornean *Nepenthes* species with differences in nitrogen acquisition strategies. Microb Ecol.

[CR56] Siragusa AJ, Swenson JE, Casamatta DA (2007). Culturable bacteria present in the fluid of the hooded-pitcher plant *Sarracenia minor* based on 16S rDNA gene sequence data. Microb Ecol.

[CR57] Sirová D, Šantrůček J, Adamec L, Bárta J, Borovec J, Pech J, Owens SM, Šantrůčková H, Schäufele R, Štorchová H, Vrba J (2014). Dinitrogen fixation associated with shoots of aquatic carnivorous plants: is it ecologically important?. Ann Bot.

[CR58] Sullivan JT, Brown SD, Ronson CW (2013). The nifA-rpoN regulon of *Mesorhizobium loti* strain R7A and its symbiotic activation by a novel LacI/GalR-family regulator. PLoS One.

[CR59] Thorén LM, Tuomi J, Kämäräinen T, Laine K (2003). Resource availability affects investment in carnivory in *Drosera rotundifolia*. New Phytol.

[CR60] Williams SE, Pickard BG (1974). Connections and barriers between cells of *Drosera* tentacles in relation to their electrophysiology. Planta.

[CR61] Yoshida K-i, Kim W-S, Kinehara M, Mukai R, Ashida H, Ikeda H, Fujita Y, Krishnan HB (2006). Identification of a functional 2-keto-*myo*-inositol dehydratase gene of *Sinorhizobium fredii* USDA191 required for *myo*-inositol utilization. Biosci Biotechnol Biochem.

[CR62] Zhang M, Lenaghan SC, Xia L, Dong L, He W, Henson WR, Fan X (2010). Nanofibers and nanoparticles from the insect-capturing adhesive of the sundew (*Drosera*) for cell attachment. J Nanobiotechnol.

