# Supplementary material for: Occurrence of myo-inositol and alkyl-substituted polysaccharide in the prey-trapping mucilage of Drosera capensis
Source: Naturwissenschaften. 2017 Sep 22;104(9):83. doi: 10.1007/s00114-017-1502-4 (PMC5610204; doi:10.1007/s00114-017-1502-4)
Supplement: Supplementary file 1 — (PDF 387 kb). [file 114_2017_1502_MOESM1_ESM.pdf]

Original Article – *The Science of Nature*

The prey-trapping mucilage of *Drosera capensis* – occurrence of *myo*-inositol, detection of lipophilic moieties on the polysaccharides, and a consideration on the reported mineral content

Tetsuo Kokubun (ORCID 0000-0001-9318-5099)

Royal Botanic Gardens, Kew, Richmond, Surrey TW9 3AB, United Kingdom

t.kokubun@kew.org

Tel: +44 (0)208 332 5365

Supplementary material

Table of contents

		Page
Figure S1	Proton NMR spectra: Isopropanol-water (8:2)-soluble fraction of *Drosera* mucilage	2
Figure S2	Carbon-13 NMR spectra: Isopropanol-water (8:2)-soluble fraction of *Drosera* mucilage	3
Figure S3	COSY spectrum. Isopropanol-water (8:2)-soluble fraction of *Drosera* mucilage	4
Figure S4	HSQC spectrum. Isopropanol-water (8:2)-soluble fraction of *Drosera* mucilage	5
Figure S5	HMBC spectrum. Isopropanol-water (8:2)-soluble fraction of *Drosera* mucilage	6
Figure S6	HSQC spectra of the polysaccharide components in *Drosera* mucilage	7
Figure S7	HMBC spectra of the polysaccharide components in *Drosera* mucilage	8
Figure S8	Proton NMR spectra of crude plant mucilages	9
Figure S9	Expansion of the aldehyde, aromatic and alkenic proton chemical shift regions	10

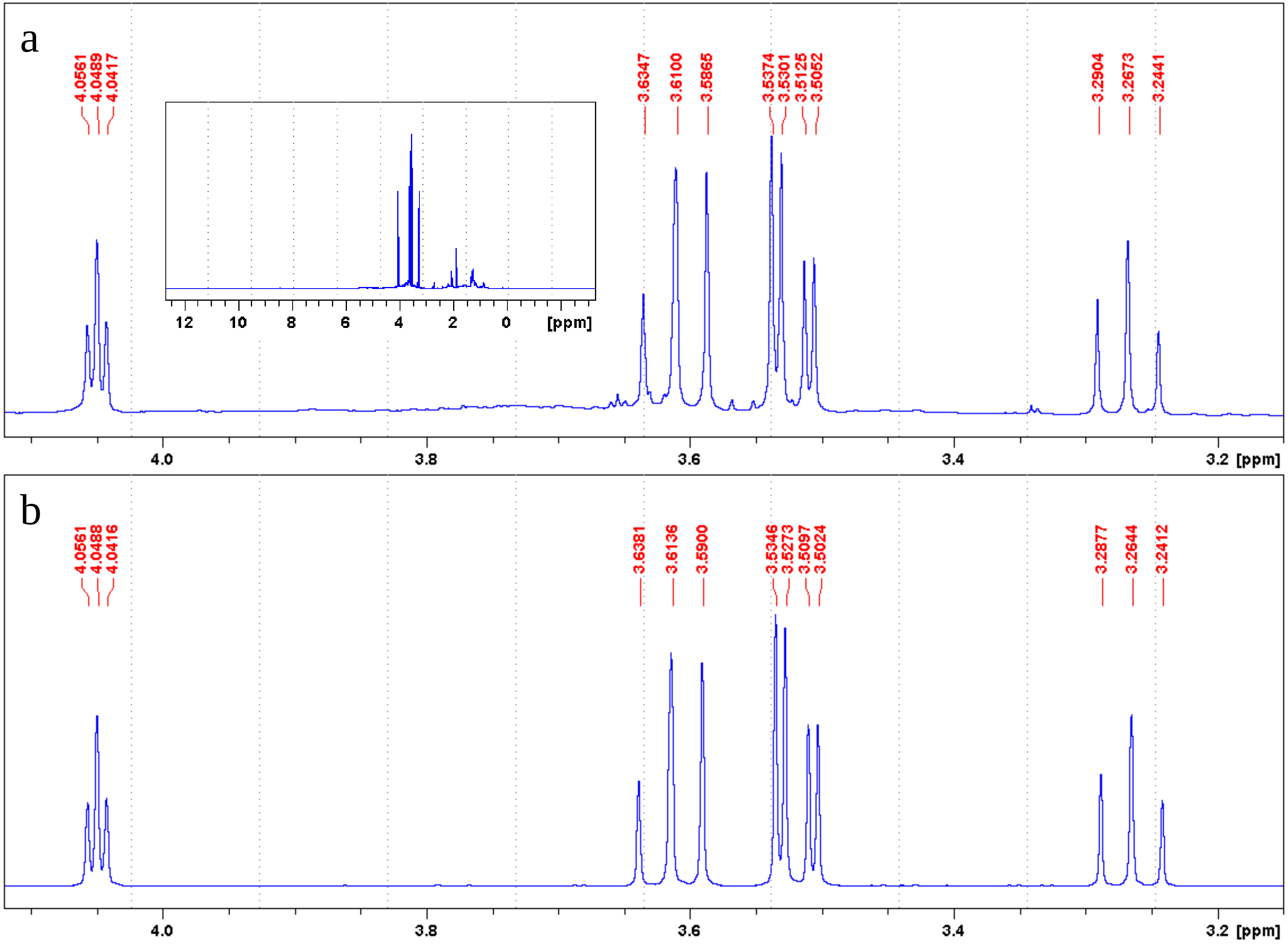

Fig. S1 Proton NMR spectra (400 MHz, D_2O, 30 °C). **a** Isopropanol-water (8:2)-soluble fraction of *Drosera* mucilage (inset: full spectrum). **b** Standard *myo*-inositol (BDH)

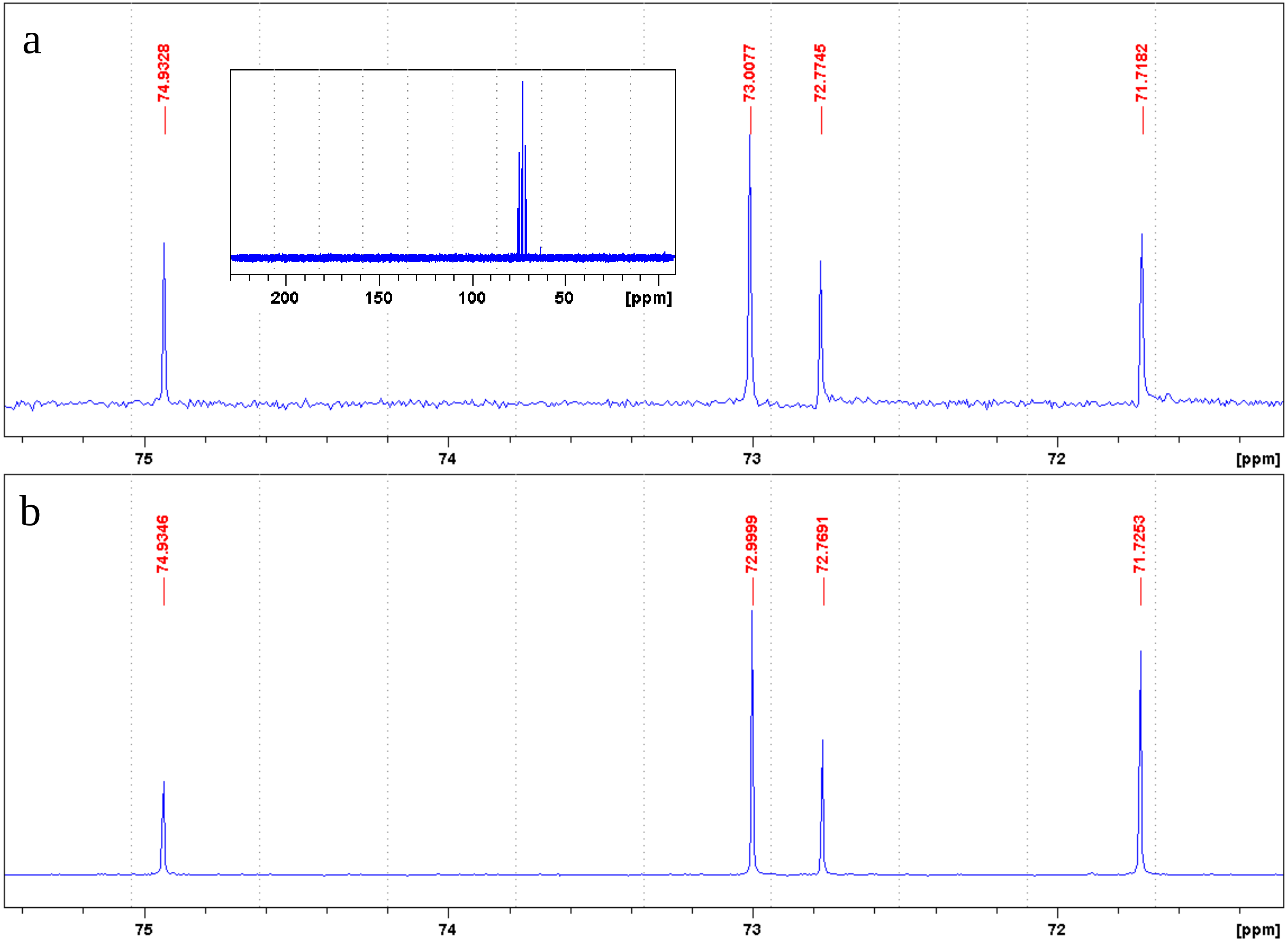

Fig. S2 Carbon-13 NMR spectra (100 MHz, D_2O, 30 °C). **a** Isopropanol-water (8:2)-soluble fraction of *Drosera* mucilage (inset: full spectrum). **b** Standard *myo*-inositol (BDH)

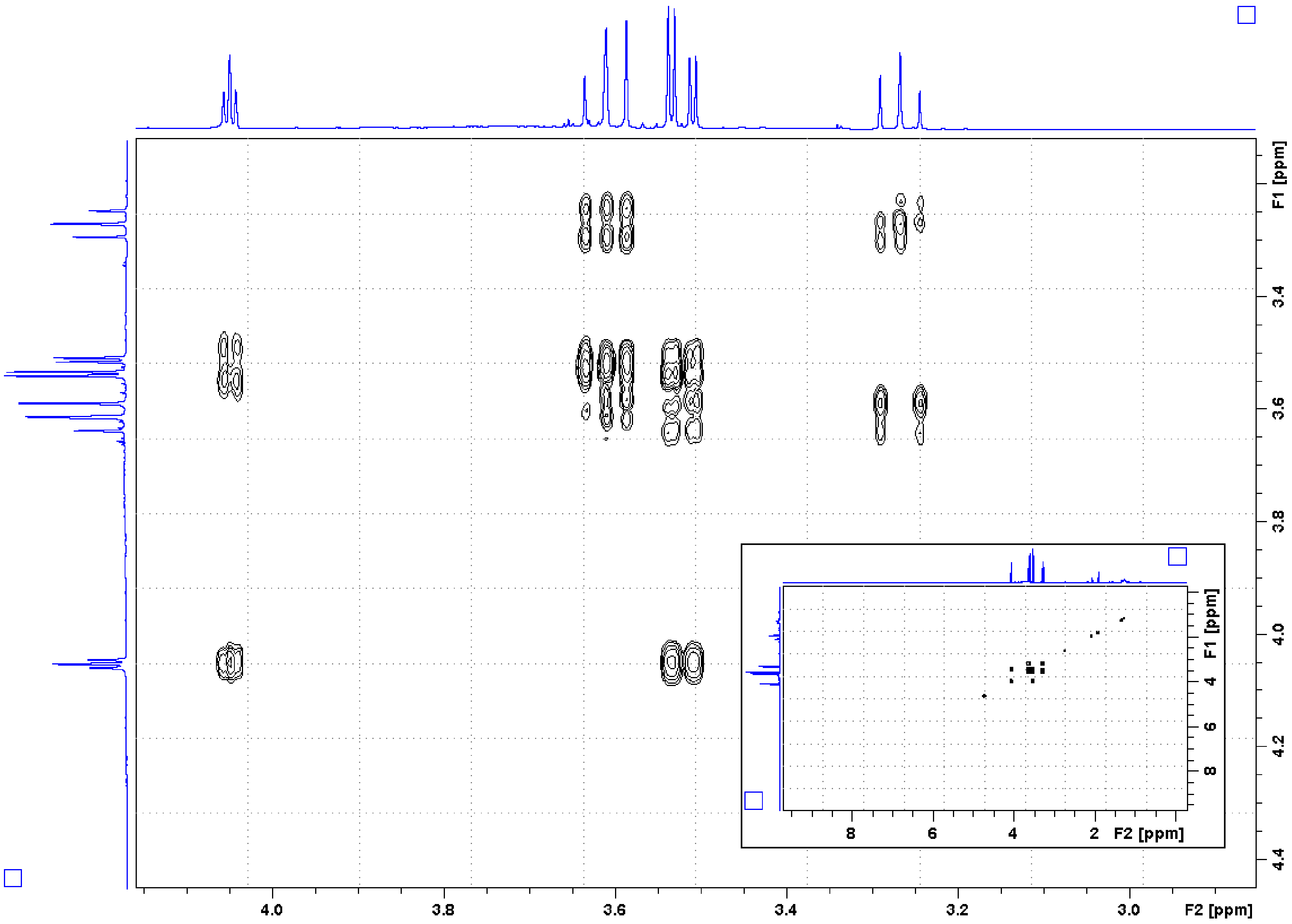

Fig. S3 COSY spectrum (400 MHz, D_2O, 30 °C). Isopropanol-water (8:2)-soluble fraction of *Drosera* mucilage (inset: full spectrum)

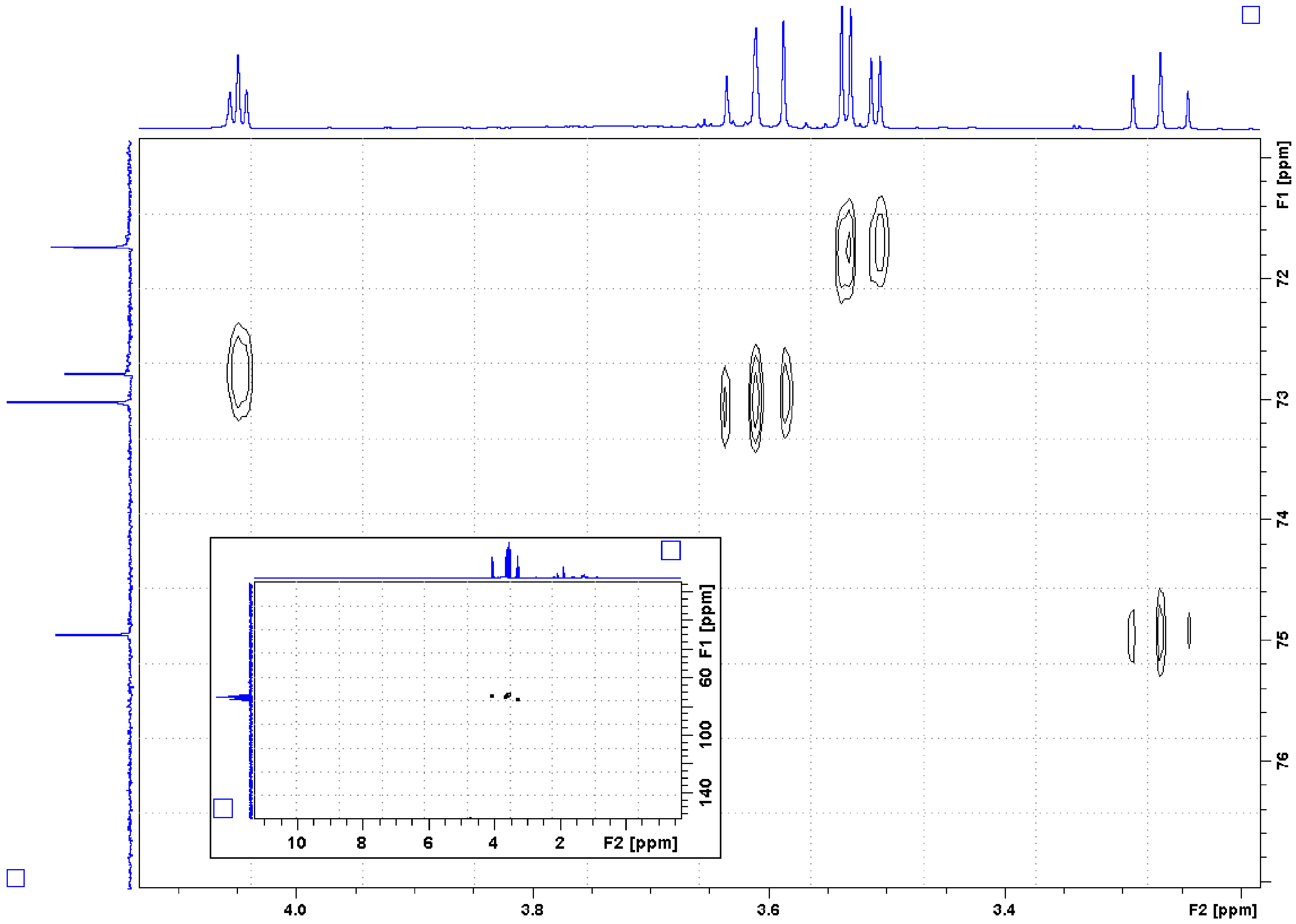

Fig. S4 HSQC spectrum (D_2O, 30 °C). Isopropanol-water (8:2)-soluble fraction of *Drosera* mucilage (inset: full spectrum)

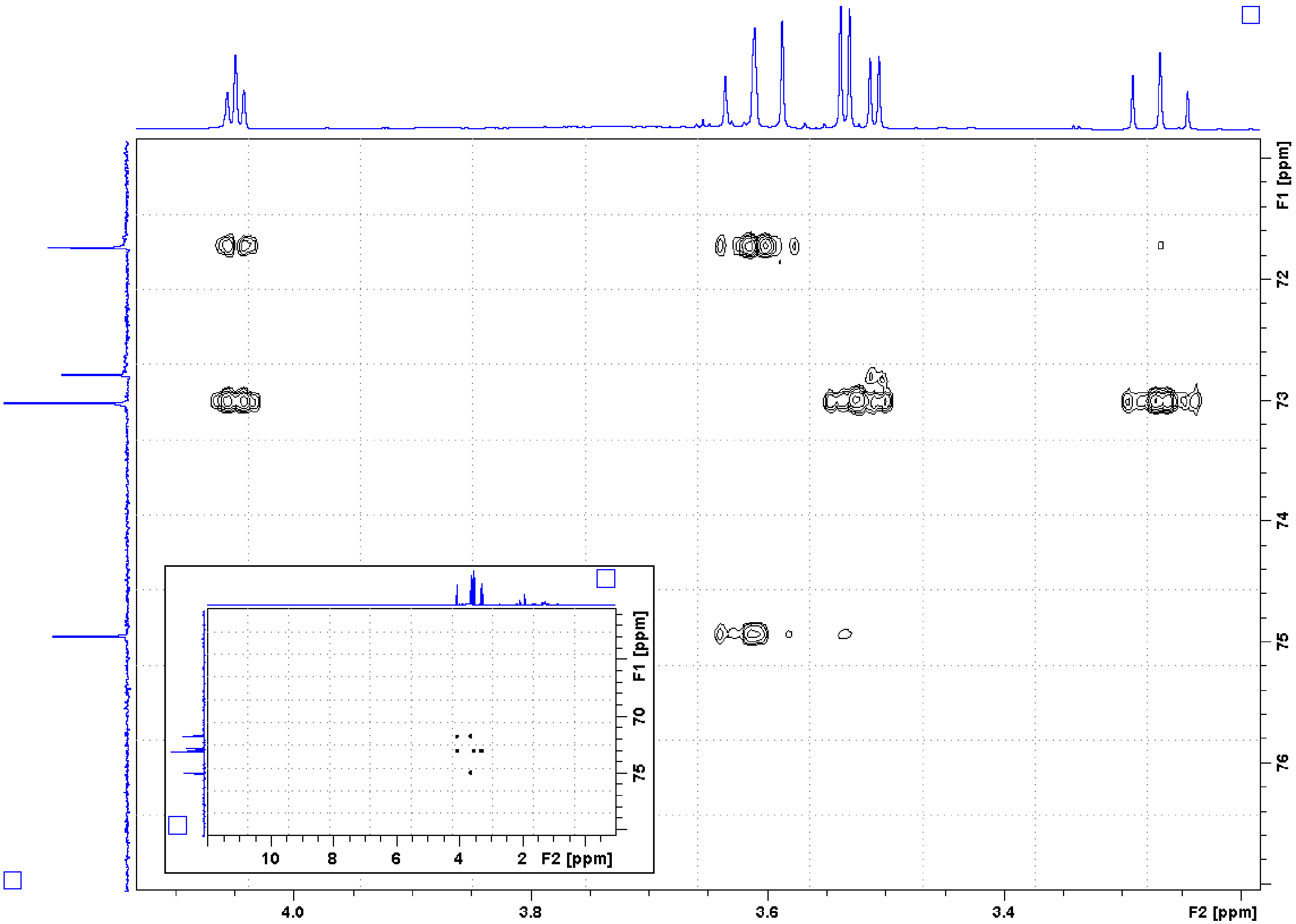

Fig. S5 HMBC spectrum (D_2O, 30 °C). Isopropanol-water (8:2)-soluble fraction of *Drosera* mucilage

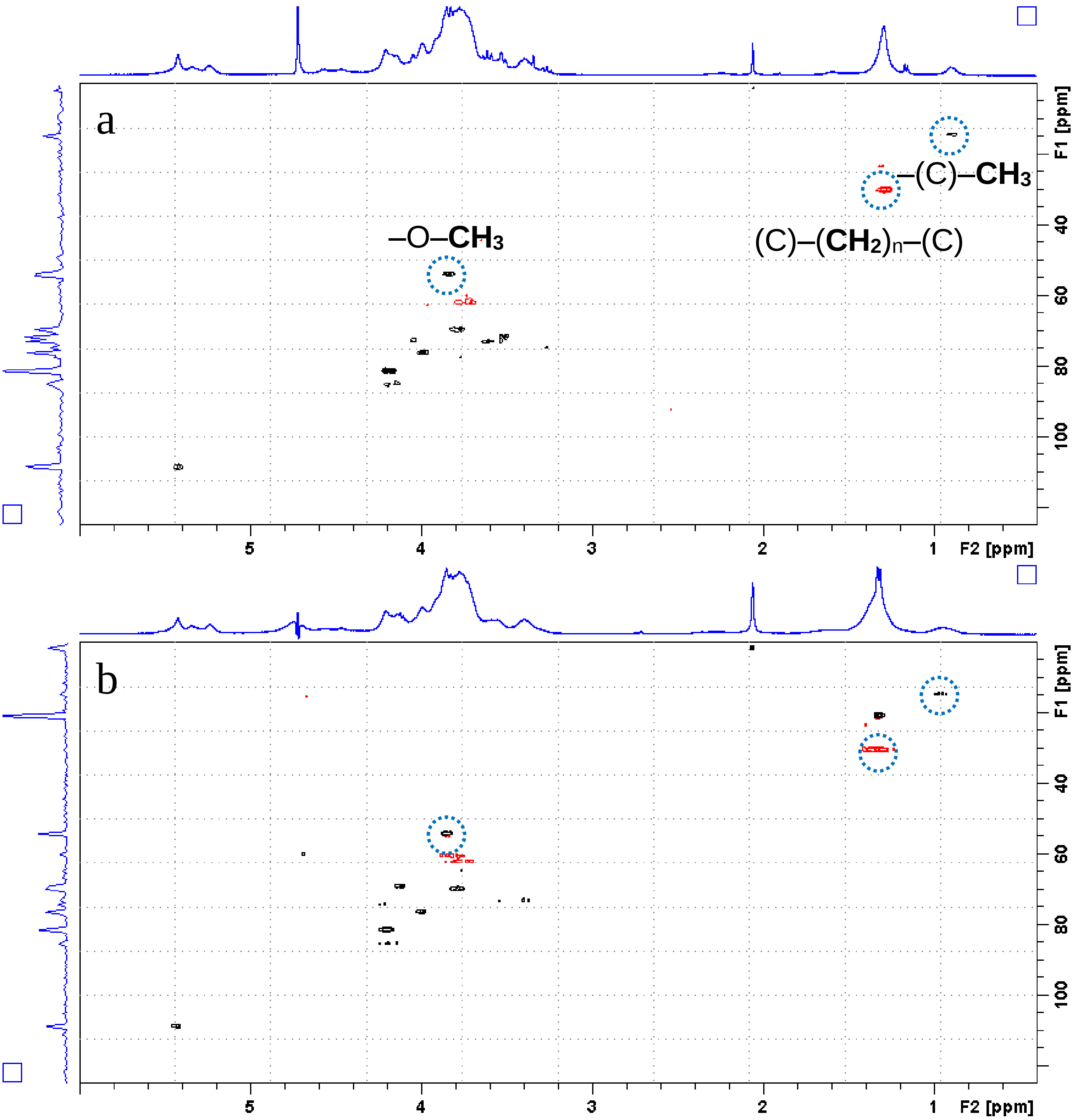

Fig. S6 HSQC spectra (D_2O, 30 °C) of the polysaccharide components in *Drosera* mucilage. **a** Isopropanol-water (8:2)-insoluble fraction. **b** Retentate of dialysis (cut-off MW 6,000–8,000). Displayed 1D proton NMR spectra (on horizontal axis) are of the respective samples, and the carbon-13 spectra are of internal (accumulated) projections of the signal intensities. Carbon-proton correlations in black are either CH_3 or CH, and those in red are CH_2 (not accumulated in the F1 axis projection)

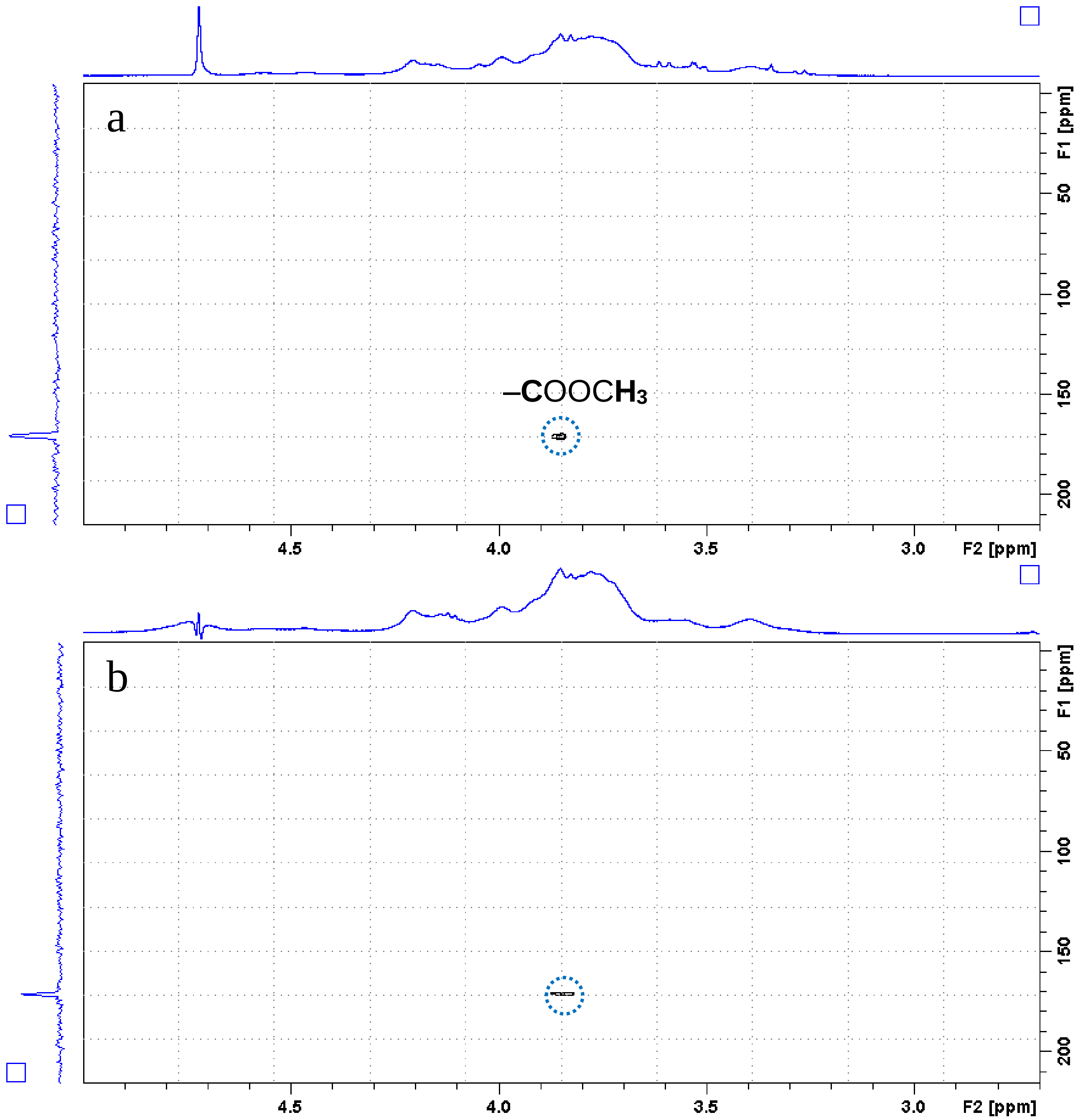

Fig. S7 HMBC spectra (D_2O, 30 °C) of polysaccharide components in *Drosera* mucilage. **a** Isopropanol-water (8:2)-insoluble fraction. **b** Retentate of dialysis (cut-off MW 6,000–8,000). Displayed 1D proton NMR spectra (on horizontal axis) are of the respective samples, and the carbon-13 spectra are of internal (accumulated) projections of the signal intensities.

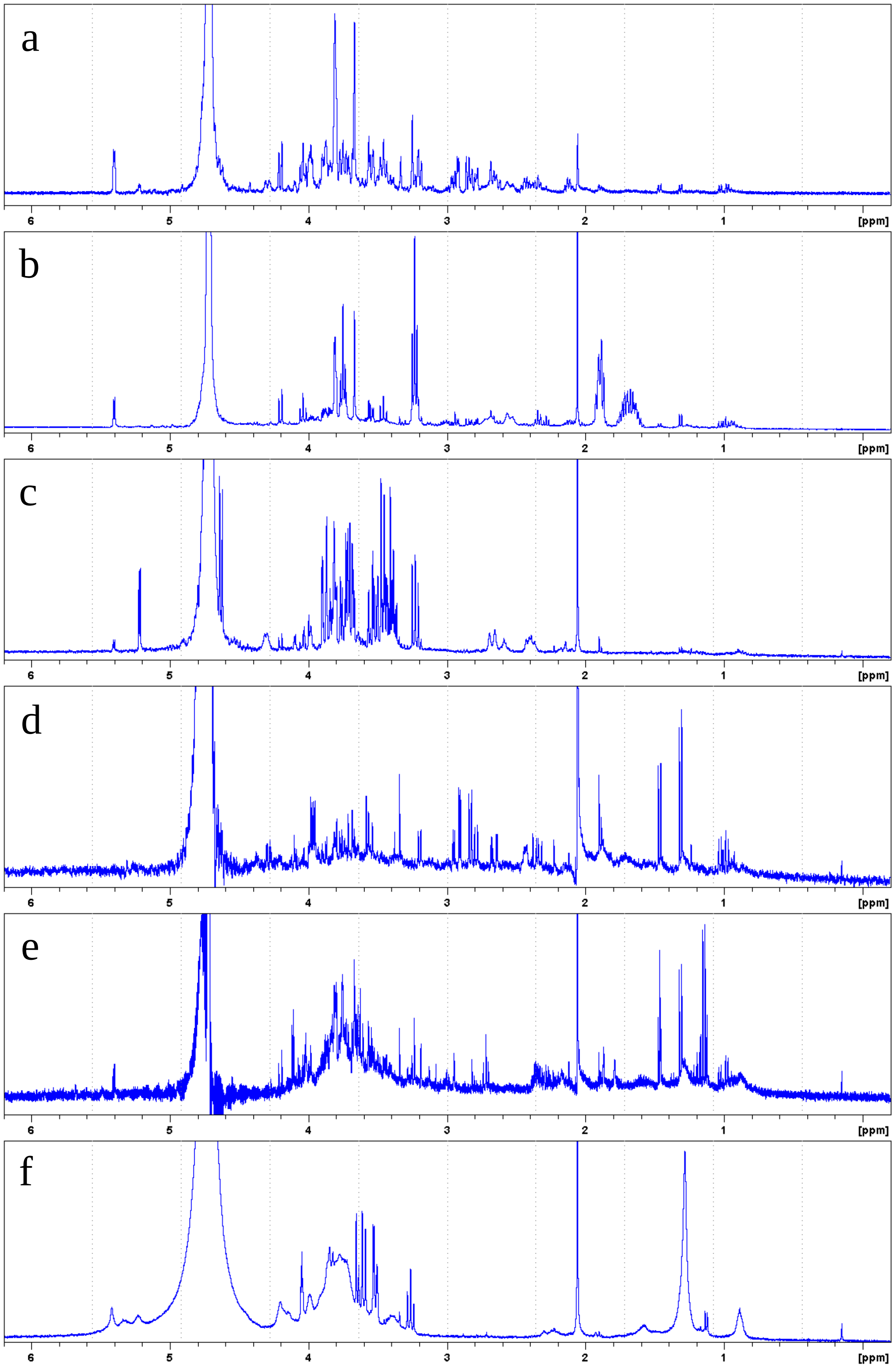

Fig. S8 Proton NMR spectra (400 MHz, D_2O, 30 °C) of crude plant mucilages. **a** *Abelmoschus esculentus* fruit (pod). **b** *Actinidia deliciosa* stem. **c** *Aloe vera* mesophyll. **d** *Lupinus polyphyllus* root tip. **e** *Zea mays* root tip. **f** *Drosera capensis* tentacle exudate (as **Fig. 1a** for comparison). Vertical axis not to scale. Chemical shifts were calibrated against the internal reference CH_3CN at 2.06 ppm

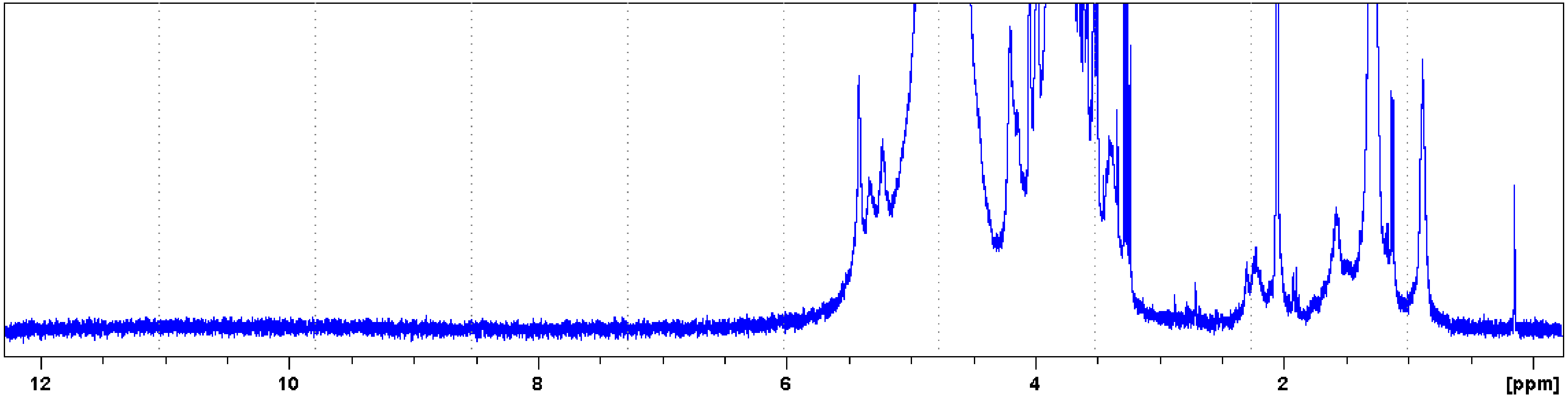

Fig. S9 Expansion of the aldehyde, aromatic and alkenic proton chemical shift regions of the NMR spectra (400 MHz, D_2O, 30 °C) of crude mucilage from *Drosera capensis* (as **Fig. 1a**). Chemical shift was calibrated against the internal reference CH_3CN at 2.06 ppm
